# Convective Mixing in Distal Pipes Exacerbates *Legionella pneumophila* Growth in Hot Water Plumbing

**DOI:** 10.3390/pathogens5010029

**Published:** 2016-03-12

**Authors:** William J. Rhoads, Amy Pruden, Marc A. Edwards

**Affiliations:** Via Department of Civil and Environmental Engineering, Virginia Tech, 1145 Perry St., 401 Durham Hall, Blacksburg, VA 24061, USA; apruden@vt.edu (A.P.); edwardm@vt.edu (M.A.E.)

**Keywords:** *Legionella pneumophila*, premise plumbing, hot water, design, installation, mixing, convection

## Abstract

*Legionella pneumophila* is known to proliferate in hot water plumbing systems, but little is known about the specific physicochemical factors that contribute to its regrowth. Here, *L. pneumophila* trends were examined in controlled, replicated pilot-scale hot water systems with continuous recirculation lines subject to two water heater settings (40 °C and 58 °C) and three distal tap water use frequencies (high, medium, and low) with two pipe configurations (oriented upward to promote convective mixing with the recirculating line and downward to prevent it). Water heater temperature setting determined where *L. pneumophila* regrowth occurred in each system, with an increase of up to 4.4 log gene copies/mL in the 40 °C system tank and recirculating line relative to influent water compared to only 2.5 log gene copies/mL regrowth in the 58 °C system. Distal pipes without convective mixing cooled to room temperature (23–24 °C) during periods of no water use, but pipes with convective mixing equilibrated to 30.5 °C in the 40 °C system and 38.8 °C in the 58 °C system. Corresponding with known temperature effects on *L. pneumophila* growth and enhanced delivery of nutrients, distal pipes with convective mixing had on average 0.2 log more gene copies/mL in the 40 °C system and 0.8 log more gene copies/mL in the 58 °C system. Importantly, this work demonstrated the potential for thermal control strategies to be undermined by distal taps in general, and convective mixing in particular.

## 1. Introduction

*Legionella pneumophila* is the most frequently reported causal agent of waterborne disease outbreaks in developed countries, causing a respiratory infection known as Legionnaires’ disease (a severe and life-threatening pneumonia) or Pontiac Fever (a milder flu-like illness that is usually self-limiting) [1,2]. Inhalation of *Legionella*-entrained aerosols is the primary exposure pathway for infection, rather than ingestion, which is the infection route for traditional fecal pathogens that are the primary basis of water regulations [3,4,5]. *L. pneumophila* serogroup 1 is estimated to have caused 84% of the 6868 reported cases of legionellosis in the United States between 2009–2010, and is thought to be the most common strain associated with disease [1,2,6,7]. Other species and serogroups can also be pathogenic, but are not as well documented [8,9].

Controlling *L. pneumophila* is a major challenge in building plumbing systems. In a recent survey, nearly half of 68 cold drinking water taps were positive for *L. pneumophila* genetic markers (272 total samples, from 25 states across the U.S.) [10]. In particular, hot water systems are a key reservoir for *Legionella* and source of disease, due to ideal growth temperatures along with dissipation of chemical disinfectant residuals [11,12,13,14,15]. The temperature dependence of *L. pneumophila* survival in hot water has been well-characterized [13,16,17,18,19,20,21]. Although some strains of *L. pneumophila* have been observed to survive brief periods exposed >70 °C water [22,23],maintaining an elevated water heater setting (>55 °C) is a widely accepted strategy for minimizing regrowth of *Legionella* [12,19,24]. However, it is important to recognize that actual water temperatures at the tap can cool considerably relative to the water heater set points [25], and maintaining desired temperatures throughout the entire plumbing system is challenging [26]. For example, distal taps experience a form of thermal shock every time hot water flows and return to ambient temperature during stagnation periods. Such conditions can actually stimulate the proliferation of *Legionella*, rather than inhibit it [25,27,28,29,30].

Water heater set points, along with plumbing configuration and water use patterns, may also have interactive effects on *L. pneumophila* proliferation. Here, we hypothesize that convective mixing induced by upward-oriented pipes and increased flow frequency at high use taps can influence the flux of nutrients to taps and the microbes that colonize there, including *Legionella*. Recent work demonstrated that assimilable organic carbon (AOC) control strategies can also be undermined by the generation of organic carbon by nitrifiers, hydrogen oxidation, and other autotrophic processes [31,32,33,34,35,36,37], and that AOC is not the master variable controlling *L. pneumophila* regrowth in a typical plumbing system [38].

Systematic, controlled studies of water heater set point on *Legionella* proliferation in plumbing are lacking, but a few available studies suggest there may be interactive effects with plumbing configuration. Culture-based surveys found that having electric water heaters and vertically oriented hot water tanks are additional risk factors for *Legionella* colonization [12,39]. Some water heaters thermally stratify with warm water on the bottom of the tank and hot water at the top, creating zones of ideal *L. pneumophila* growth temperature [40,41]. With respect to pipe orientation and convective mixing specifically, thermal gradients in otherwise stagnant water have been documented to exacerbate pipe corrosion under cold water pipe conditions between 5–25 °C [42], but have never been studied with respect to possible impacts on microbial communities. In hot water systems, the potential for convective mixing is high given the stark temperature differential between hot water in tanks and recirculating lines (up to 60 °C) and ambient room temperature influencing stagnant pipes (20 °C). Water in downward oriented distal pipes (e.g., pipes from a recirculating hot water loop located in the ceiling running downwards to distal taps) will not convectively mix [42]. However, we hypothesize that horizontal and upward oriented distal pipes will convectively mix with hot water in recirculating loops, warming the otherwise stagnant water in the distal lines and enhancing flux of nutrients and corresponding regrowth of microorganisms (Table 1) [42,43].

**Figure 1:**
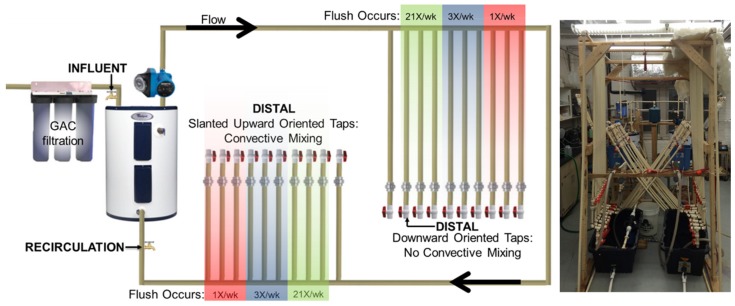
Overview of experimental design of replicated building plumbing systems. Convective mixing was induced in upward oriented pipes by slanting each pipe 30° from vertical, whereas downward oriented pipes provide a control without convective mixing. One plumbing system tank and recirculating line was maintained at 40 °C (Warm Temperature) while the other was maintained at 58 °C (Hot Temperature) over 4 months. Influent water was flushed through three granular activated carbon whole-house filters (Sample port: Influent), a recirculating pump continuously pumped water around the return loop back to the water heater creating a completely mixed reservoir (Sample port: Recirculation), six replicate distal taps (three upward + three downward) were flushed at 3.8 L/min (1 gallon/min) 21×/week, 3×/week, and 1×/week for a total of 36 pipes (Sample ports: at end of distal pipes).

Herein we report the first controlled, large-scale, replicated laboratory examination of interrelationships between water heater temperature set point, convective mixing within distal plumbing, and distal tap use frequency on *L. pneumophila* occurrence and regrowth. Identical “Warm Temperature Set Point” and “Hot Temperature Set Point” hot water plumbing systems were constructed in which continuously recirculating pipe loops delivered water to two sets of distal taps subject to high, medium, and low water use frequency. One set of pipes was oriented to promote convective mixing with the recirculating loop while the other set was oriented to prevent convective mixing (Figure 1). The Warm Temperature system was set to 40 °C and the Hot Temperature system to 58 °C. Genetic markers of *Legionella* spp. (23S rRNA gene), *L. pneumophila* (macrophage infectivity potentiator (*mip*) gene), *Vermamoeba vermiformis* (18S rRNA gene; an important ecological host for *Legionella*), and total bacteria (16S rRNA gene) were tracked by quantitative polymerase chain reaction (q-PCR) to measure regrowth in the recirculating lines and distal taps.

## 2. Results and Discussion

After describing the effects of convective mixing on temperature profiles in distal taps, its effect on other physicochemical trends including chlorine, ammonia, and total organic carbon (TOC) are presented. Thereafter, regrowth and occurrence of *Legionella* (*L. pneumophila* and *Legionella* spp.) and other ecologically relevant microorganisms is examined as a function of water use patterns and convective mixing (Table 1). The discussion is focused on behavior of target organisms measured in water flushed from the system, as this is the source of consumer exposure, but biofilm results are included as well. An overview of the calculations used in this study to compare the distribution of *L. pneumophila* between the Hot and Warm Temperature systems, and across their various niches, are provided in the supplementary materials (Table S1).

### 2.1. Convective Mixing in Distal Taps.

**Figure 2:**
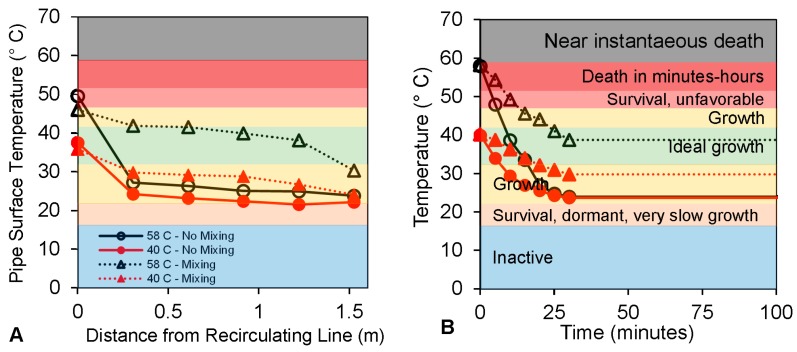
Temperature profiles in the Warm and Hot Temperature systems. Temperature profiles with and without convective mixing (**A**) along the length of distal pipes during periods of no water use (pipe surface temperature measured with infrared emissivity probe, each point represents average of *n* = 9) and (**B**) within pipes as a function of time immediately after flushing (each data point represents temperature of a ~100 mL first flush sample collected from individual distal taps at the time indicated after all distal pipes were flushed with water from the recirculating line); shaded regions on the plots illustrate temperature dependent ranges of *Legionella* growth [17,18,19,20,21,22,23].

The two experimental systems were aged 15 months without significant convective mixing of water in distal taps as previously reported elsewhere [25]. After the initial 15 month period, convective mixing was initiated in one set of distal taps by orienting the pipe 30° off vertical, significantly altering the temperature profile of water in the pipes [43]. During periods of stagnation, distal pipes without convective mixing cooled to room temperature while distal pipes with convective mixing equilibrated to warmer temperatures. The measured pipe surface temperature profiles along the length of distal taps when water was not being used confirmed convective mixing in upward oriented pipes, maintaining higher temperature throughout the pipes (Figure 2A). There was no evidence of convective mixing in downward oriented pipes, as pipes cooled to room temperature at distances less than 30 cm from the recirculation line (Figure 2A). Notably, the last 1 meter of distal pipes with convective mixing were 5–12 °C warmer in the Hot Temperature system than pipes that did not convectively mix when water was not being used. Convective mixing also altered temperature profiles within the distal taps immediately following water use. Water temperatures in otherwise stagnant distal pipes without convective mixing cooled to ambient room temperature (23–24 °C) within 30 min after use in both systems, but equilibrated in distal pipes that did convectively mix to 30.5 °C in the Warm system and to 38.8 °C in the Hot system (Figure 2B). Convective mixing was stronger in the Hot Temperature system because it had a larger temperature differential between the water heater set point and ambient room temperature. In general, the entire volume of the Warm Temperature system water heater and recirculating line were consistently maintained at 40.6 °C, near the upper limit of the ideal growth range for *L. pneumophila*, while water in the distal taps was in the ideal growth range only up to 3% of the time due to cooling during stagnation (Table 2). In contrast, the hot water heater and recirculating line were maintained in the ideal growth range 0% of the time, while the distal taps were in the ideal growth range at least 95% of the time due to convective mixing (Table 2). The entire length of pipe would likely equilibrate at higher temperatures if more conductive pipe materials (e.g., copper) or insulation had been used, which would have different implications for bacterial growth.

### 2.2. Other Physicochemical Trends

#### 2.2.1. Total Chlorine

Influent chloramine disinfectant residual was reduced by three granular activated carbon (GAC) filters in series. Average total chlorine concentration in the influent water was always less than 0.10 mg Cl_2_/L; thus, achieving the goal of isolating the effects of convective mixing, temperature setting, and water use pattern, which are the focus of this study.

#### 2.2.2. Ammonia (NH_3_) Oxidation and Total Organic Carbon (TOC) Production

**Figure 3:**
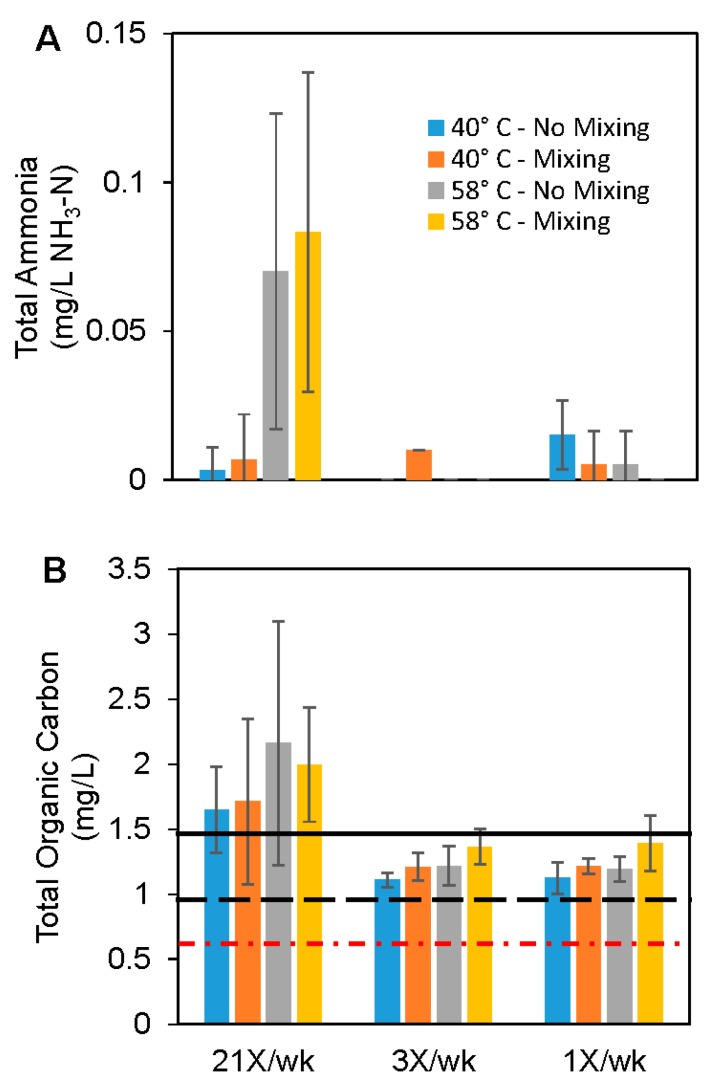
Ammonia and total organic carbon trends in tank and distal taps. (**A**) Average ammonia (NH_3_-N) in distal taps. Influent water had 0.56 mg/L NH_3_-N, the Hot Temperature system had 0.27 mg/L NH_3_-N at the end stagnation periods, and Warm Temperature system had 0.0 mg/L NH_3_-N at the end stagnation periods; (**B**) Average total organic carbon (TOC) concentration after regular stagnation period between flushes (*i.e.*, immediately before flushing occurs). NH_3_ and TOC concentrations are indicated in the 40 °C system tank and recirculating line (solid black line; note ammonia in the 40 °C tank and recirculating line is 0 mg/L), in the 58 °C system tank and recirculating line (dashed blackline), and in the influent water (dashed-dot red line). Error bars represent 95% confidence intervals (*n* = 2–6).

In the Warm Temperature system, 100% (0.56 mg/L NH_3_-N) of the influent ammonia was oxidized in the tank and recirculating line reservoir between distal pipe flushes, indicating the presence of active nitrifying microorganisms [33,37]. In the Hot Temperature system, only 52% of the total influent ammonia was oxidized between flushes in the tank and recirculating lines, likely because nitrifying bacteria reduce in activity above 45–55 °C [44,45,46]. In fact, at these high temperatures, it is possible that nitrification was not occurring in the main tank and recirculating line, but mainly in the distal taps where temperature was cooler and 100% of the ammonia delivered by convective mixing was oxidized in medium and low use distal taps (Figure 3A). In that case, the ammonia drop in the main tank could be due to convective exchange with the distal taps. (Figure 3A). Assuming this is the case, we estimated that the convective mixing exchange rate (net convectively driven flow of water from the recirculation line to the distal tap during stagnation) would need to be about 0.14 L/h to achevie the measured steady state ammonia concentration of 0.27 ± 0.07 mg/L NH_3_-N observed in the tank (Supporting Microsoft Excel Workbook). A potassium tracer, dosed into the experimental tank and monitored at the distal taps over time by taking very small (~20 mL) aliquot samples, resulted in an estimated water exchange rate of 0.12 L/h, which is very close to our estimate.

Total organic carbon (TOC) increased by a factor of 2.7 (from 0.54 mg/L in the filtered influent water to 1.47 mg/L in the tank and recirculating line) in the Warm Temperature system, presumably due to autotrophic growth. In addition to nitrifying bacteria using residual ammonia from chloramines, hydrogen oxidizing bacteria may also have contributed to the increase, by using hydrogen created from corrosion of the water heater sacrificial anode rod [40]. There was relatively little further increase of TOC in the high use distal taps, but in the medium and low use taps TOC decreased by 0.26–0.39 mg/L relative to the recirculating line, presumably due to microbial uptake for growth and respiration. Convective mixing in upward oriented pipes did not have a significant effect on the TOC, with only 0.09–0.13 mg/L more TOC than pipes that did not mix (Figure 3B; Paired *t*-Test, *p*-value = 0.47, *n* = 18). In contrast, TOC increased by only a factor of 1.8 (from 0.54 mg/L to 0.96 mg/L) in the Hot Temperature tank and recirculating line, presumably due to lower levels of autotrophic growth as illustrated by the lower levels of ammonia removal noted previously. The 1.1–1.3 mg/L increase in TOC observed in the high use pipes relative to the recirculating line may reflect the high activity of autotrophs in these taps, and the smaller increases in TOC in medium and low use pipes could indicate greater uptake by heterotrophic bacteria. Pipes with convective mixing generally had more TOC than pipes without convective mixing (0.07–0.2 mg TOC/L), except the Hot Temperature system high use taps (which had 0.16 mg TOC/L less) (Paired *t*-Test, *p*-value = 0.70, *n* = 18). In general, the trends observed in the data were qualitatively consistent with our hypothesis (Table 1), even though they were not generally statistically significant. Given that our system had ample nutrients (nitrogen and organic carbon), convective mixing could be playing only a small role in regrowth potential in these systems, but may be increasingly important in systems with lower nutrients, as mixing in those cases might deliver a higher fraction of total nutrients relative to that supplied during flushing.

### 2.3. General Trends in Bulk Water Legionella 

*L. pneumophila* levels in the Warm Temperature system recirculating line and distal taps remained stable after fifteen months of aging and over the four month timeframe of this experiment (Figure 4; Figure S1). In our prior study that took place during the 6–15 month window of operation, baseline levels of *L. pneumophila* steadily increased in the recirculating line from 5.0 × 10^3^ to 1.6 × 10^4^ copies/mL and in the medium and low use distal taps from 1.6 × 10^4^ to 1.3 × 10^5^ copies/mL [25]. In the present study, *L. pneumophila* was below the quantification limit in the cold, filtered influent water while the recirculating line of the Warm system remained at 2.51 × 10^4^–6.31 × 10^4^ gene copies/mL. There was no change from these values in the high use distal pipes and only a 0.1 log decrease to a 0.6 log increase in gene copies/mL in low and medium use distal pipes (Figure 4c). In other words, the vast majority of the *L. pneumophila* growth occurred within the tank and recirculating line of the Warm Temperature system (Figure 4a). The highest total yield of *L. pneumophila* was from the highest use taps (Figure 4b), given that concentration is multiplied by frequency of use (Table S1) [25]. In the Warm Temperature system, the high concentrations of *L. pneumophila* that regrew in the tank and recirculating lines were delivered to the distal taps with each use. Under such conditions, orientation of the distal taps and use frequency had little effect on levels of *L. pneumophila* at each tap.

Consistent with our hypothesis, the elevated temperature setting in the Hot Temperature system produced much lower populations of *L. pneumophila* in the tank and recirculating line, but the benefits of the elevated temperature setting did not fully translate to the distal pipes (Tables S2 and S3) [25]. In general, there was a 1.6–3.5 log decrease in total yield of *L. pneumophila* in the Hot Temperature system compared to corresponding pipe conditions in the Warm Temperature system (Figure 4b). However, convective mixing in distal pipes in this work reduced the effectiveness of the elevated temperature. For instance, in low use taps without convective mixing, the elevated temperature in the Hot Temperature system decreased *L. pneumophila* levels by an average of 2.3 logs compared to the Warm Temperature system. However, in low use pipes with mixing, the elevated temperatures only reduced *L. pneumophila* levels by an average of 1.6 logs (Figure 4a; Mann-Whitney U-Test, *p* = 0.020, n _mixing_ = n _no mixing_ = 6). Similar trends were observed with medium and high use taps (Figure 4a). In addition, the only case where low use taps had a greater total *L. pneumophila* yield than high use taps occurred in the Hot Temperature system pipes with convective mixing, with 1.2 log more total weekly yield of *L. pneumophila* than high use taps (Figure 4b, 4 month sampling). Clearly, in the Hot Temperature system, orientation of the distal taps and frequency of use were major factors in determining the levels of *L. pneumophila* at each tap.

*Legionella* spp. occurrence was also of interest, given that there are more than 50 species and 70 serogroups of *Legionella*, and that many besides *L. pneumophila* are also pathogenic. Interestingly, *Legionella* spp. was not as highly impacted by the elevated temperature settings in the Hot Temperature system as *L. pneumophila* (Figure 4b and Figure S2). In general, total yield of *Legionella* spp. was reduced up to only 1.5 logs in the Hot *versus* Warm Temperature system, compared to the 3.5 log decrease in *L. pneumophila* under some conditions (Figure S2b). However, in the Hot Temperature system, low use pipes had substantially more *Legionella* spp. regrowth (increasing to 4.7–5.1 log gene copies/mL from 3.6 log gene copies/mL in the recirculating line) than in medium or high use pipes (increasing to only 3.4–4.7 log gene copies/mL from 3.6 log gene copies/mL in the recirculating line). Similar to our previous work [23], the elevated operating temperature of the Hot Temperature system significantly decreased the ratio of *L. pneumophila* compared to *Legionella* spp., from 0.42 to 0.05 (Paired *t*-Test, *p*-value < 0.0001, *n* = 36). Thus, higher temperatures in this experiment appeared to select other *Legionella* spp. relative to *L. pneumophila.*

### 2.4. Impact of Convective Mixing on Bulk Water Legionella

A direct comparison of *Legionella* spp. and *L. pneumophila* in pipes with mixing to pipes without mixing yields 12 comparisons (3 use frequencies × 2 temperature settings × 2 samplings = 12).

Convective mixing increased *L. pneumophila* regrowth in both the Hot and Warm Temperature systems in 11 of 12 conditions (4 of the 11 were statistically higher based on 95% confidence intervals; Figure 5). In the one condition where pipes without convective mixing had higher *L. pneumophila* concentrations than pipes with mixing, there was one outlier in the triplicate pipes without mixing that may have skewed the results (Figure S1). The sharpest increases due to convective mixing (3 of the 4 significant conditions) occurred in the low and medium use pipes, consistent with the hypothesis that the convective mixing gradients help supply ideal growth temperatures and nutrients in the Hot Temperature system. In fact, the low use distal taps with convective mixing in the Hot Temperature system had only 0.9–1.6 log fewer *L. pneumophila* than the recirculating line of the Warm Temperature system. This is expected since this small volume of Hot Temperature low use distal tap water was effectively mixed during stagnation and held at ideal temperature, much as is the case in the Warm Temperature tank and recirculating line (Figure 4a).

*Legionella* spp. occurrence was not as strongly impacted by convective mixing gradients as *L. pneumophila* (Figure 5). *Legionella* spp. concentrations were elevated in pipes with mixing compared to pipes without mixing in 8 of 12 conditions (5 of those 8 were statistically higher based on 95% confidence intervals). However, the conditions with significant increases were distributed between all three use frequencies and both temperature settings, producing no clear trend. Convective mixing also tended to decrease the proportion of *L. pneumophila* relative to all *Legionella* spp. in the Warm Temperature system distal taps (by 0.14–0.26) and increase it in the Hot Experimental system distal taps (by 0.013–0.15) (Figure S5). In these same conditions (Warm and Hot Temperature system distal taps with convective mixing), *L. pneumophila* and *Legionella* spp. were significantly, albeit weakly, correlated (Spearman’s Rank ρ = 0.37–0.57, *p*-value = 0.005–0.013). In the Warm Temperature system, convective mixing supplied the distal taps with an influx of diverse and active microorganisms, potentially reducing the competitiveness of *L. pneumophila* and providing nutrients for all *Legionella* spp. to regrow. Hence, we observed weak correlations between *L. pneumophila* and *Legionella* spp. In the Hot Temperature system, convective mixing continuously inoculated distal taps with a less active microbial community with respect to *Legionella* growth compared to the Warm Temperature system, along with unused nutrients from the recirculating line, and were maintained at ideal growth temperatures. This apparently resulted in regrowth of all *Legionella* spp., but favored *L. pneumophila.* Thus, *Legionella* spp. and *L. pneumophila* were better correlated in the Hot Water system distal taps and the relative ratio of *L. pneumophila* slightly increased compared to pipes with no convective mixing. However, both systems initially had high levels of total bacteria genetic markers in the recirculating lines and tank (7.2–7.7 log gene copies/mL), suggesting a shift in the microbial community instead of inhibiting regrowth altogether. Convective mixing also increased total bacteria in distal pipes relative to pipes without mixing (with 0.1–0.7 log more 16S rRNA gene copies/mL). Overall, this study suggests that there is variable response across *Legionella* spp. to water stressors experienced in hot water plumbing. While many species share the ability to infect hosts, and even share specific virulence genes, pathogenesis is differentiated among *Legionella* spp., which may extend to survival and growth mechanisms within plumbing systems as witnessed here [47,48,49,50,51,52,53].

### 2.5. Trends in Biofilm Legionella 

To provide a measurement of biofilm re-colonization, the same area (65 cm^2^) was swabbed in each reservoir/distal tap during each experimental period. At the end of our previous study [25], there were 4.4 × 10^5^ gene copies/cm^2^ in the system maintained at approximately 40 °C. In the Warm Temperature system, the recirculating lines had the greatest *L. pneumophila* biofilm densities (up to 1.3 × 10^4^ gene copies/cm^2^), again consistent with continuous recirculation and delivery of nutrients to biofilms at ideal temperature (Figure 6). In the distal taps at cooler temperatures*, L. pneumophila* did not recolonize the biofilm to the same extent after the previous sampling, having 0.4–2.5 log fewer *L. pneumophila* gene copies/cm^2^ than the recirculating line (Figure 6; Figure S3). High use distal taps tended to have slightly higher densities of *L. pneumophila* recolonize the biofilm after each sampling compared to less frequently used taps (0.1–0.5 log more gene copies/cm^2^ in 3 of 4 conditions). Pipes with convective mixing tended to have higher densities recolonize the biofilm than pipes without mixing (0.70–1.27 log more gene copies/cm^2^); however, neither trend had statistically significant differences. Similar trends occurred with respect to *Legionella* spp. (Figure S4). These findings support the hypothesis that the majority of regrowth occurred in the recirculating line of the Warm Temperature system, where all the ammonia was consumed and the majority of the TOC was produced.

*L. pneumophila* appeared unable to recolonize the biofilm after each resampling in the Hot Temperature system. The one condition where recolonization was observed (2.5 log gene copies/cm^2^ in the 2 month sampling of the high use pipes with mixing) had significant regrowth in only one of three triplicate pipes (Figure S3b). More regrowth would be expected if the entire distal pipe was maintained at ideal growth temperatures instead of the gradient we observed (Figure 2A), as might be the case if pipe insulation or heat-conducing pipe (e.g., copper) had been used. Thus, the repeated swabbing of a small portion of the system surface provides insights into the ability of the system to re-establish a biofilm rapidly. More regrowth was expected considering that the bulk water *L. pneumophila* results were consistent with the hypotheses in Table 1. However, these results do indicate that the use of elevated temperatures reduce the likelihood of *L. pneumophila* colonization if temperatures are maintained at hot enough levels to inhibit regrowth, which is difficult to achieve and maintain in complex plumbing networks [24].

### 2.6. Ecological Relationships

**Figure 7:**
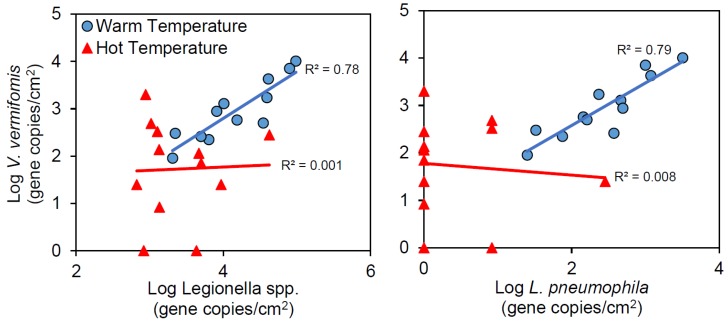
Correlation of *V. vermiformis* with (**A**) *Legionella* spp. and (**B**) *L. pneumophila* in distal tap biofilms.

*Legionella* proliferation in drinking water systems is widely thought to be facilitated by growth within a host amoeba cell, with a broad range of host organisms having been described. Here, we focus on biofilm-associated *V. vermiformis* because it is among the most frequently detected host organisms in drinking water [54,55,56], was found to be the most prevalent amoeba and was weakly correlated to *Legionella* spp. in a prior investigation of Blacksburg, VA tap water [57], and amoeba primarily graze on organisms inhabiting biofilms. In the Warm Temperature system tank and recirculating lines, high levels of *V. vermiformis* were detected (average 1.1 × 10^4^ gene copies/cm^2^) relative to the influent (3.2 × 10^3^ gene copies/cm^2^), as were high levels of *Legionella* spp. (2.36 × 10^3^ gene copies/cm^2^), *L. pneumophila* (8.2 × 10^3^ gene copies/cm^2^), TOC production (0.93 mg/L increase), and ammonia oxidation (0.54 mg/L consumed). Thus, under these ideal conditions, all indicators of regrowth were in agreement. In the distal taps, which received high levels of all bacteria and maintained favorable regrowth conditions, *V. vermiformis* strongly correlated with *Legionella* spp. and *L. pneumophila* (Figure 7a,b; R^2^ = 0.78–0.79). However, in the Hot Temperature system, there were no *V. vermiformis* detected in the recirculating line and tank, and no correlations were found in the distal taps with *Legionella* spp. or *L. pneumophila* (Figure 7a,b; R^2^ = 0.001–0.008). In addition, there were 1.2 log fewer *V. vermiformis* gene copies/cm^2^ in the distal taps of the Hot Temperature system than the Warm Temperature system on average (paired *t*-test, *p*-value = 0.0011, *n* = 72). In the Warm Temperature system, the strong correlations of *Legionella* and *V. vermiformis* are consistent with the expectation of the pathogen-host relationship. However, in the Hot Temperature system, the elevated temperatures may have caused *V. vermiformis* to encyst, resulting in decreased phagocytosis of *Legionella* cells and decreased recovery of the amoebae genetic material in general. Interestingly, low use distal taps in the Hot Temperature system had higher concentrations of *V. vermiformis* than high or medium use taps (681 *vs*. 92 gene copies/mL, Mann-Whitney U-test, *p*-value = 0.023). This suggests the week long stagnation in the low use distal taps was sufficient for the amoebae to recover from the encysted phase that may have been induced by the elevated temperature in the Hot Temperature system. Finally, convective mixing in the Warm Temperature system was associated with an increase in the number of *V. vermiformis* detected (paired *t*-test, *p*-value = 0.025, *n* = 36) and improved correlations with *Legionella* spp. (R^2^ = 0.71 without mixing *vs*. 0.99 with mixing), but not with *L. pneumophila* (R^2^ = 0.76 without mixing vs 0.80 with mixing). No such trends were found in the bulk water (Figure S6).

### 2.7. Limitations of Experimental Conditions

Despite the consistent impact of convective mixing in distal pipes that occurred in this study, which facilitated additional growth of *Legionella* spp. and *L. pneumophila* relative to distal pipes without convective mixing, it is important to note that under other operating conditions the opposite effect is expected to occur. For instance, in later experiments using the same experimental systems, we introduced a 0.42 ± 0.05 mg/L (as total Cl_2_) chloramine residual to both water heaters. After a typical stagnation period, chloramine concentrations in the recirculating lines were 0.28 ± 0.08 mg/L. In low use downward oriented taps with no convective mixing, the residual decayed to 0.06 ± 0.02 mg/L while pipes with convective mixing maintained 0.13 ± 0.02 mg/L of chloramine delivered from the recirculating line. Under this scenario, convective mixing would be expected to decrease the ability for *Legionella* and *L. pneumophila* to regrow relative to pipes without convective mixing due to the increased presence of chloramine [57,58,59]. Similarly, factors that impact the convective water exchange rate between the recirculating line and distal taps (*i.e.*, pipe diameter, insulation, conductive copper *vs.* non-conductive plastics) may also create different trends than results observed in this study.

In addition, there are inherent limitations to the methodology applied in this study. While qPCR provides quantitative insights into the presence and increase in gene targets, it does not insure only live cells are quantified. Culturing data could have provided insight into the species and strains of *Legionella* that grow on isolation media; however, the viable but non-cultivable state of *Legionella* makes definitive conclusions over the dominant species difficult.

## 3. Experimental Section

### 3.1. Experimental Setup and Operation

Details of the experimental system design were previously published in a study examining the effect of water heater set points without convective mixing [25]. Briefly, two identical household hot water systems were constructed with 71.9 L (19 gallon) electric water heaters and continuously recirculating pipe loops (Figure 1). For this work, the “Warm Temperature Set Point” system water heater was set to 40 °C and the “Hot Temperature Set Point” system water heater was set to 58 °C to represent worst and best management practices with respect to *Legionella* control. Both systems were acclimated for 15 months with no convective mixing prior to commencing this study (month 15 in our previous work is month 0 for this study) [25]. For this study, convective mixing was induced in half the distal pipes by slanting upward oriented pipes 30° from vertical. Sampling occurred at 2 months and 4 months after inducing convective mixing to compare the two pipe orientations (with and without convective mixing) with three water use patterns in triplicate, including low use (1 flush/week), medium use (3 flushes/week), and high use (21 flushes/week) for a total of 36 distal taps (2 systems × 2 orientations × 3 use patterns × triplicate = 36) to simulate the range of use frequencies encountered in homes. Each distal pipe was 1.5 m long with a 0.2 m section accessible by a union for repeated biofilm swab, had a volume of approximately 430 mL, and each flush described above was 28 seconds long at 3.8 L/min (1 gallon/min). Influent water consisted of well-flushed (10 min at 11.3 L/min), GAC filtered Blacksburg, VA tap water. During periods of no use, distal pipes were allowed to equilibrate with the ambient temperature. All pipes were uninsulated.

### 3.2. Sample Collection and Microbiological Analyses

At the end of each two-month period, approximately 0.5 L of first-flush water was collected directly from the influent, recirculating lines, and each distal tap at the end of regular stagnation periods for each use condition. Samples were filtered through sterile 0.22 µm pore-size mixed cellulose ester filters (Millipore, Billerica, MA, USA). Exact volume collected for each sample was measured by weight after filtration. For biofilm sampling, 65 cm^2^ of influent, recirculating line, and ends of the distal tap pipes accessible by threaded union connections were swabbed using sterile cotton-tip applicators (Fisherbrand, Fisher Scientific, UK). DNA was extracted directly from fragmented filters and cotton swabs using a FastDNA Spin Kit (MP Biomedicals, Solon, OH, USA) according to the manufacturer protocol. Field, trip, and equipment negative controls consisting of pre-sterilized (autoclaved) nanopure water in identical sampling bottles were included each time samples were collected.

Gene markers for *Legionella* spp. (23S rRNA), *L. pneumophila* (macrophage infectivity potentiator (*mip*) gene), *V. vermiformis* (18S rRNA), and total bacteria (16S rRNA gene), were enumerated by q-PCR assays using previously established methods [57]. In brief, all q-PCR assays were performed in 10 μL reaction mixtures containing SsoFast Probes or Evagreen Supermix (Bio-Rad, Hercules, CA, USA), 250 or 400 nM primer, 93.75 nM probe (Taqman assay only) with 1 μL of DNA template. For each set of samples, serial dilutions (ranging from 1:1 to 1:100) with positive template spikes were used to identify the optimum dilution to minimize q-PCR inhibition (1:10) on a subset of samples. A negative control, 10-fold serial dilutions of standards, and a positive spike into sample DNA matrix were included in triplicate wells with each q-PCR run. The quantification limit (QL) for all q-PCR assays ranged from 10 to 100 gene copies/reaction, except 16S rRNA which ranged from 200–1000 gene copies/reaction. The QL was applied to samples based on standard curves obtained on each thermocycler run. Samples yielding threshold cycles ≥ QL in at least two q-PCR triplicate wells were considered quantifiable. Samples with only one triplicate above the QL threshold cycle, or samples otherwise below the QL were re-analyzed undiluted to increase the QL of the assays. On each re-run plate, standard DNA template was spiked into the experimental DNA matrix to confirm amplification reactions were not inhibited in undiluted samples. If inhibited, the sample was marked as below the QL. All values are reported as log(gene copies/mL + 1).

Disinfectant residual, total ammonia, temperature, pH, dissolved oxygen (DO), total organic carbon (TOC), and total and dissolved cations were generally characterized in the first week of each 2-month operation period. Once trends were established, disinfectant, ammonia, temperature, and pH were monitored to confirm parameters were not significantly changing. Chloramine and total ammonia were measured according to Standard Method 4500-Cl25 and 5310-NH3 using a DR2700 or DR5000 spectrophotometer (HACH, Loveland, CO, USA). pH and temperature were measured using a pH 110 meter with automatic temperature correction (Oakton Research, Vernon Hills, IL, USA). Pipe surface temperature was measured using an infrared emissivity temperature probe (Kintrex Infrared Thermometer IRT0421). Pipe surface profiles were quantified by measuring the pipe surface temperature at 0.3 m (1 ft) intervals at the end of regular stagnation periods. Dissolved Oxygen was monitored using a Thermo Scientific Orion 3-star meter. Total organic carbon was measured by persulfate-ultraviolet detection using a Sievers Model 5300C with an autosampler according to Standard Method 5310 C. Cations were measured by inductively coupled plasma mass spectrometry after acidification with 2% nitric acid (v/v) and >24 h holding time.

### 3.3. Statistical Analyses

All error bars on figures and error margin calculations are 95% confidence intervals, calculated based on the normal cumulative distribution function, degrees of freedom, and standard error. For graphing and statistical purposes, any positive detection below the q-PCR QL was entered as half of the lowest observed QL. All data analysis was conducted in Microsoft Excel 2013, JMP Pro 11, or RStudio using R version 3.2.0. Spearman’s rank coefficient and associated significance tests were conducted in JMP Pro 11 to detect and quantify relationships between gene markers (using “Multivariate Methods”). Other statistical tests were performed in RStudio. Student’s *t*-test (“t.test()”) and Kruskal-Wallis tests with a Holm p-value adjustment for multiple comparisons were conducted to compare sample means (initially with “kruskal.test(),” then using package and function “dunn.test()” for multiple comparisons). Significance was determined at *p* = 0.05.

## 4. Conclusions

Here we examined the interactive effects of convective mixing induced by plumbing configuration along with water heater temperature setting and water use frequency on the growth of *Legionella* spp. and *L. pneumophila* in recirculating lines and otherwise stagnant distal pipes. This large pilot-scale experiment supports the conventional wisdom that maintaining elevated water temperatures at all points in a hot water system is a critical engineering control for inhibiting regrowth of *Legionella,* especially *L. pneumophila*. This work demonstrated that convective mixing currents in hot water systems can maintain ideal growth temperatures and continuously supply nutrients to otherwise stagnant distal taps. As a result, convective mixing has the potential to undermine thermal control strategies. In these experiments, the benefits of maintaining water above 55 °C in the water heater and recirculating line were reduced in the distal taps, and convective mixing facilitated additional *L. pneumophila* regrowth. Thus, holistic approaches are needed to control *L. pneumophila* regrowth with attention to subtle design considerations that can inadvertently impact the extent of regrowth. Other factors expected to alter convective mixing exchange between reservoirs in hot water plumbing (such as pipe diameter, use of insulation, and use of conductive metallic pipe) are expected to affect regrowth at distal taps.

## Figures and Tables

**Figure 4:** Heat map of bulk water *L. pneumophila*. Heat map of bulk water *L. pneumophila* compares (**a**) concentration in the tank and recirculating lines and each set of distal taps (log gene copies/mL); (**b**) total yield of *L. pneumophila* per week at the tap (log gene copies); and (**c**) log change in concentration in distal taps with respect to the recirculating lines (regrowth factor). Colors are on a continuous scales from green (low) to red (high). Table S1 provides a detailed description of each calculation. “*” indicates calculations done with estimated values that were below the quantification limit. BQL indicates “below quantification limit.”

**Table:** 

**(a) Log average (range) *L. pneumophila* concentration (gene copies/mL)**								
Condition I: Water Heater Set to 40 °C					Condition II: Water Heater Set to 58 °C			
Time	Orientation	Low	Medium	High	Orientation	Low	Medium	High
2 months	Tank & Recirc	4.8 (4.7–4.9)			Tank & Recirc	2.5 (0–3.0)		
	Distal (No Mixing)	4.9 (4.5–5.1)	4.7 (4.4–4.8)	4.8 (4.6–5.0)	Distal (No Mixing)	2.4 (2.3–2.5)	BQL	1.7 (BQL-2.0)
	Distal (w/Mixing)	5.0 (4.6–5.1)	5.3 (5.2–5.4)	4.8 (4.7–4.9)	Distal (w/Mixing)	3.2 (3.0–3.3)	3.7 (3.6–3.9)	3.1 (3.1)
4 months	Tank & Recirc	4.4 (4.4)			Tank & Recirc	0.0		
	Distal (No Mixing)	4.9 (4.8–5.0)	4.5 (4.5–4.6)	4.5 (4.5)	Distal (No Mixing)	2.8 (2.6–3.0)	2.7 (0–3.0)	2.0 (0–2.47)
	Distal (w/Mixing)	5.0 (4..7–5.3)	4.9 (4.4–5.2)	4.6 (4.5–4.6)	Distal (w/Mixing)	3.5 (3.4–3.6)	2.9 (0–3.2)	BQL
**(b) Log total average *L. pneumophila* weekly yield (gene copies)**								
Condition I: Water Heater Set to 40 °C					Condition II: Water Heater Set to 58 °C			
Time	Orientation	Low	Medium	High	Orientation	Low	Medium	High
2 months	Distal (No Mixing)	7.6	7.9	8.8	Distal (No Mixing)	5.1	4.4*	5.7
	Distal (w/ Mixing)	7.7	8.5	8.8	Distal (w/ Mixing)	5.9	6.9	7.0
4 months	Distal (No Mixing)	7.6	7.7	8.5	Distal (No Mixing)	5.5	5.9	6.0
	Distal (w/ Mixing)	7.7	8.1	8.6	Distal (w/ Mixing)	6.2	6.0	4.9*
**(c) Log average change in *L. pneumophila* in distal taps relative to recirculating lines (gene copies/mL)**								
	Condition I: Water Heater Set to 40 °C				Condition II: Water Heater Set to 58 °C			
Time	Orientation	Low	Medium	High	Orientation	Low	Medium	High
2 months	Distal (No Mixing)	0.1	−0.1	0.0	Distal (No Mixing)	−0.1	−1.3	−0.8
	Distal (w/ Mixing)	0.2	0.5	0.0	Distal (w/ Mixing)	0.6	1.2 *	0.4
4 months	Distal (No Mixing)	0.5	0.1	0.1	Distal (No Mixing)	2.8	2.7	2.0
	Distal (w/ Mixing)	0.6	0.5	0.2	Distal (w/ Mixing)	3.5	2.9	0.9 *

**Figure 5:** Log difference in bulk water *Legionella* spp. and *L. pneumophila* concentration in pipes with convective mixing compared to pipes without convective mixing. Numbers near zero indicate no change between pipes with and without convective mixing; numbers near to 1 or −1 indicate a 10× increase or decrease in concentration in pipes with convective mixing compared to pipes without convective mixing. Positive numbers indicate that pipes with convective mixing have greater concentrations than pipes with no convective mixing. Bold numbers are statistically significant (Kruskal-Wallis Test, *p* < 0.05). * indicates calculations carried out with estimated values that were below the quantification limit.

**Table:** 

	Water Heater Set to 40 °C			Water Heater Set to 58 °C		
Condition	Low	Medium	High	Low	Medium	High
***Legionella* spp.**						
2 months	**0.55**	**0.79**	0.30	0.07	**0.89**	**0.61**
4 months	0.02	**0.45**	0.20	0.37	−0.23 *	0.29
***L. pneumophila***						
2 months	0.11	**0.60**	0.02	**0.76**	**2.51**	1.27
4 months	0.12	0.37	0.06	**0.73**	0.17	−1.08 *

**Figure 6:** Heat map of *L. pneumophila* biofilm (log gene copies/cm^2^) comparing concentration in the tank and recirculating line biofilm to each set of distal taps. Colors are on a continuous scales from green (low) to red (high). BQL indicates values that were detectable, but below the quantification limit. ND indicates there were no genes detected.

**Table:** 

Log average *L. pneumophila* concentration (gene copies/cm^2^)								
	Condition I: Water Heater Set to 40 °C				Condition II: Water Heater Set to 58 °C			
Condition	Orientation	Low	Medium	High	Orientation	Low	Medium	High
2 months	Recirculating Line	3.9			Recirculating Line	ND		
	Distal (No Mixing)	2.2 (2.1-2.3)	2.2 (BQL-2.3)	2.4 (1.4-2.6)	Distal (No Mixing)	ND	ND	ND
	Distal (w/ Mixing)	3.0 (2.7-3.2)	3.1 (2.7-3.3)	3.5 (2.8-3.8)	Distal (w/ Mixing)	ND	BQL	2.5 (BQL-2.9)
4 months	Recirculating Line	4.1			Recirculating Line	BQL		
	Distal (No Mixing)	1.9 (BQ-2.4)	BQL	BQL	Distal (No Mixing)	BQL (ND-BQL)	ND	ND
	Distal (w/ Mixing)	2.6 (2.4-2.7)	2.7 (2.5-2.9)	2.7 (2.4-2.8)	Distal (w/ Mixing)	ND	BQL (ND-BQL)	ND

**Table 1:** Hypothesized effects of convective mixing on *Legionella* spp. and *L. pneumophila* in stagnant distal taps under Warm (40 °C) and Hot (58 °C) operating conditions.

Experimental Condition	Water in Tank and Recirculating Line	Impact of Convective Mixing in Distal Taps	Net effect of Mixing on Regrowth in Distal Taps
I. No disinfectant and warm water heater set point (T = 40 °C).	Active microbial community with high levels of *L. pneumophila* in tank and recirculating line due to continuous flow of nutrients at ideal growth T	Very weak convective mixing due to low ∆T delivers some nutrients and slightly increases temperature	Marginally more regrowth in taps subject to convective mixing, but dwarfed by regrowth in tank and recirculating line
II. No disinfectant and hot water heater set point (T = 58 °C)	Thermophilic microbial community with low levels of *L. pneumophila* due to consistent inhibitory T	Stronger mixing due to greater ∆T delivers nutrients and maintains temperatures closer to ideal growth range	High concentrations in low use convectively mixed taps due to warm temperatures, continuous nutrient delivery, and sufficient regrowth time

T = Temperature.

**Table 2:** Percent of overall time the tank/recirculation lines or distal taps were maintained at temperatures suitable or ideal for *Legionella* growth; ranges represent differences resulting from different water use conditions.

System	System Volume	Ideal Growth (32–42 °C)	Growth (22–32 °C)	Limited Growth (>50 °C)
Warm Temperature Set Point	Tank+Recirc Line	100	0	0
	Mixing Distal Pipes	0.2–3.1	96.9–99.9	0
	Non-Mixing Distal Pipe	0.05–1.0	99.0–99.9	0
Hot Temperature Set Point	Tank+Recirc Line	0	0	100
	Mixing Distal Pipe	94.8–99.8	0	0.1–2.1
	Non-Mixing Distal Pipe	0.05–1.0	96.9–99.9	0.04–0.8

## Supplementary Materials

The following are available online at www.mdpi.com/2076-0817/5/1/29/s1.
