# Supplementary material for: Convective Mixing in Distal Pipes Exacerbates Legionella pneumophila Growth in Hot Water Plumbing
Source: Pathogens. 2016 Mar 12;5(1):29. doi: 10.3390/pathogens5010029 (PMC4810150; doi:10.3390/pathogens5010029)
Supplement: Supplementary file 1 [file pathogens-05-00029-s001.zip › Rhoads et al_Pathogens_Supplemantery Materials.pdf]

Supplemantery Materials: Convective Mixing in Distal Pipes Exacerbates *Legionella pneumophila* Growth in Hot Water Plumbing

William J. Rhoads, Amy Pruden and Marc A. Edwards

Table S1. Example calculations for determining distribution of genetic markers across various system compartments and effects of operating conditions.

Analysis	Calculation	Practical Meaning/Interpretation
Concentration (Figure 4A)	$log_{10}([Lp])$	*L. pneumophila* concentration
Weekly yield (Figure 4B)	$log_{10}([Lp]_{Distal} \times \forall_{Distal} \times Use)$	Total amount of *L. pneumophila* delivered at the tap per week
Change in Distal (Figure 4C)	$log_{10}\left(\frac{[Lp]_{Distal}}{[Lp]_{Recirc}}\right)$	Regrowth in the distal taps relative to the recirculating line
Convective Mixing Impact (Figure 5)	$log_{10}\left(\frac{[Lp]_{Mixing}}{[Lp]_{No\ Mixing}}\right)$	Increase in *L. pneumophila* in pipes due to convective mixing gradients

Notes: [Lp]—*L. pneumophila* concentration in gene copies/mL; Distal—"in Distal Tap"; Recirc—"in Recirculating line and tank"; $\forall$ —"Volume"; Mixing—Pipes with convective mixing; No Mixing—Pipes without convective mixing; Use—Use per week (21, 3, or 1).

Table S2. Average total number of bulk water *Legionella* spp. gene copies in each reservoir during each sampling (for each sampling, *n* = 18 for Distal Taps; *n* = 2 for Tank & Recirc).

System	Reservoir	2 Months	4 Months
Water Heater Set to 40° C	Distal Taps	1.62×10^9	2.15×10^9
	Tank & Recirc	6.53×10^9	1.25×10^{10}
Water Heater Set to 58° C	Distal Taps	3.74×10^8	3.35×10^8
	Tank & Recirc	2.93×10^8	2.99×10^8
**Log increase in 39 °C System normalized to 58 °C System:**		**40 °C–58 °C**	**40 °C–58 °C**
Distal tap *Legionella* spp. genes		0.6	0.8
Tank+Recirc *Legionella* spp. genes		1.3	1.6
**Total *Legionella* spp. genes**		**1.1**	**1.4**

Table S3. Average total number of bulk water *L. pneumophila* gene copies in each reservoir during each sampling (for each sampling, *n* = 18 for Distal Taps; *n* = 2 for Tank & Recirc).

System	Reservoir	2 Months	4 Months
Water Heater Set to 40 °C	Distal Taps	6.83×10^8	2.26×10^8
	Tank & Recirc	4.67×10^9	1.77×10^9
Water Heater Set to 58 °C	Distal Taps	1.02×10^7	1.66×10^6
	Tank & Recirc	2.50×10^7	0.00
**Log increase in 40 °C System normalized to 58 °C System:**		**40 °C–58 °C**	**40 °C–58 °C**
Distal tap *L. pneumophila* genes		1.8	2.1
Tank & Recirc *L. pneumophila* genes		2.3	9.2
**Total system *L. pneumophila* genes**		**2.2**	**3.1**

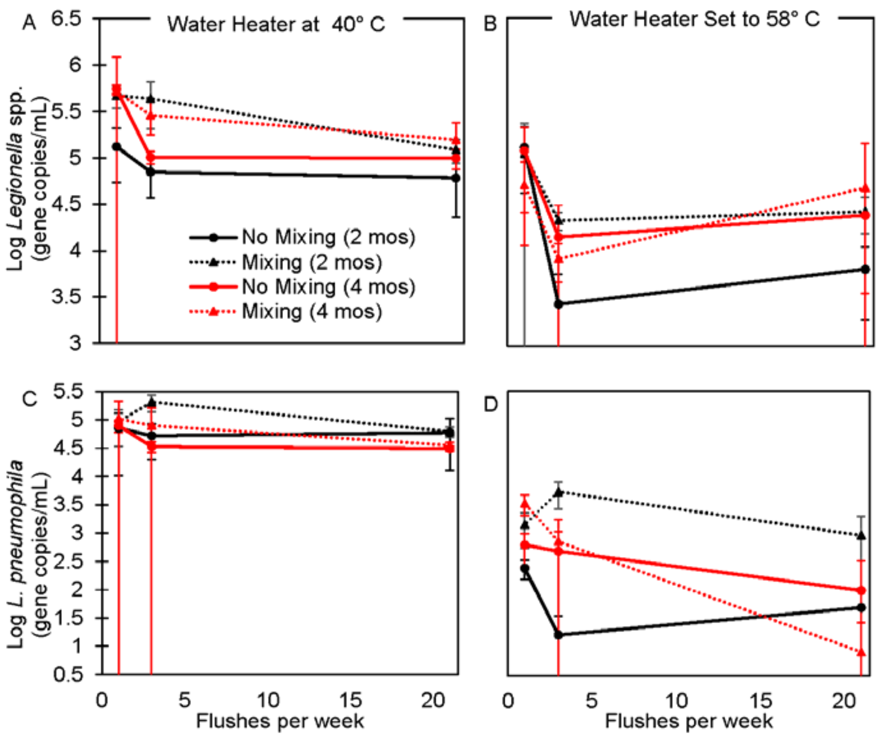

Figure S1. *Legionella* spp. and *L. pneumophila* concentrations in distal tap water when water heater is set to (**A**) and (**C**) 40 °C and (**B**) and (**D**) 58 °C.

**(a) Average Log *Legionella* spp. concentration (gene copies/mL)**								
	Condition I: Water Heater Set to 40° C				Condition II: Water Heater Set to 58° C			
Time	Orientation	Low	Medium	High	Orientation	Low	Medium	High
2 months	Tank & Recirc	4.9			Tank & Recirc	3.6		
	Distal (No Mixing)	5.1	4.8	4.8	Distal (No Mixing)	5.1	3.4	3.8
	Distal (w/Mixing)	5.7	5.6	5.1	Distal (w/Mixing)	5.0	4.3	4.4
4 months	Tank & Recirc	5.2			Tank & Recirc	3.6		
	Distal (No Mixing)	5.7	5.0	5.0	Distal (No Mixing)	5.1	4.2	4.4
	Distal (w/Mixing)	5.7	5.5	5.2	Distal (w/Mixing)	4.7	3.9	4.7
**(b) Average Log total *Legionella* spp. weekly yield from distal taps (gene copies)**								
	Condition I: Water Heater Set to 40° C				Condition II: Water Heater Set to 58° C			
Time	Orientation	Low	Medium	High	Orientation	Low	Medium	High
2 months	Distal (No Mixing)	7.8	8.0	8.8	Distal (No Mixing)	7.8	6.6	7.8
	Distal (w/Mixing)	8.4	8.8	9.1	Distal (w/Mixing)	7.7	7.5	8.5
4 months	Distal (No Mixing)	8.4	8.2	9.0	Distal (No Mixing)	7.8	7.3	8.4
	Distal (w/Mixing)	8.4	8.6	9.2	Distal (w/Mixing)	7.4	7.1	8.7
**(c) Average Log change in *Legionella* spp. in distal taps relative to recirculating lines (gene copies/mL)**								
	Condition I: Water Heater Set to 40° C				Condition II: Water Heater Set to 58° C			
Time	Orientation	Low	Medium	High	Orientation	Low	Medium	High
2 months	Distal (No Mixing)	0.2	-0.1	-0.2	Distal (No Mixing)	1.5	-0.2	0.2
	Distal (w/ Mixing)	0.7	0.7	0.1	Distal (w/ Mixing)	1.4	0.7	0.8
4 months	Distal (No Mixing)	0.5	-0.2	-0.2	Distal (No Mixing)	1.5	0.6	0.8
	Distal (w/ Mixing)	0.5	0.2	0.0	Distal (w/ Mixing)	1.1	0.3	1.1

Figure S2. Heat map of planktonic *Legionella* spp. comparing (**a**) concentration in the tank & recirculating lines and each set distal taps (average log gene copies/mL); (**b**) total yield of *L. pneumophila* per week at the tap (average log gene copies); and (**c**) average log change in concentration in distal taps with respect to the recirculating lines (regrowth factor). Colors are on a continuous scales from green (low) to red (high).

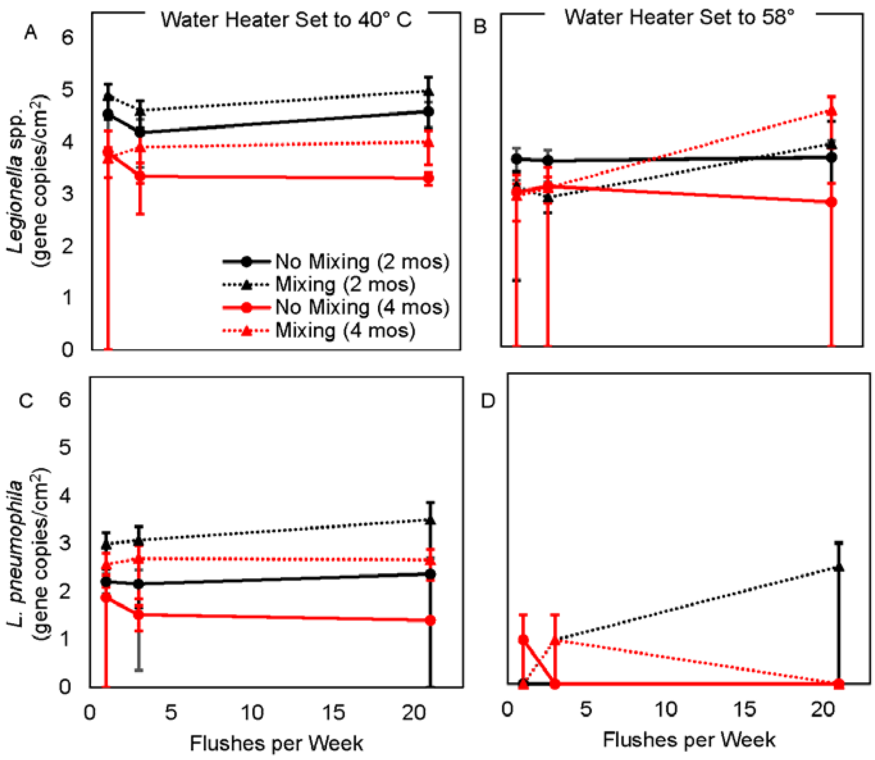

Figure S3. Average log *Legionella* spp. and *L. pneumophila* concentrations in distal tap biofilms when water heater is set to (**A**) and (**C**) 40 °C and (**B**) and (**D**) 58 °C.

	Condition I: Water Heater Set to 40° C				Condition II: Water Heater Set to 58° C			
**Condition**	**Orientation**	**Low**	**Medium**	**High**	**Orientation**	**Low**	**Medium**	**High**
2 months	Recirculating Line		5.4		Recirculating Line		3.7	
	Distal (No Mixing)	4.5	4.2	4.6	Distal (No Mixing)	3.7	3.6	3.7
	Distal (w/ Mixing)	4.9	4.6	5.0	Distal (w/ Mixing)	3.1	2.9	4.0
4 months	Recirculating Line		4.6		Recirculating Line		1.4	
	Distal (No Mixing)	3.8	3.3	3.3	Distal (No Mixing)	3.0	3.1	2.8
	Distal (w/ Mixing)	3.7	3.9	4.0	Distal (w/ Mixing)	2.9	3.1	4.6

Figure S4. Heat map of *Legionella* spp.comparing concentration in the tank & recirculating line biofilm to each set distal tap biofilm (average log gene copies/cm^2). Colors are on a continuous scales from green (low) to red (high). BQL indicates values that were detectable, but below the quantification limit.

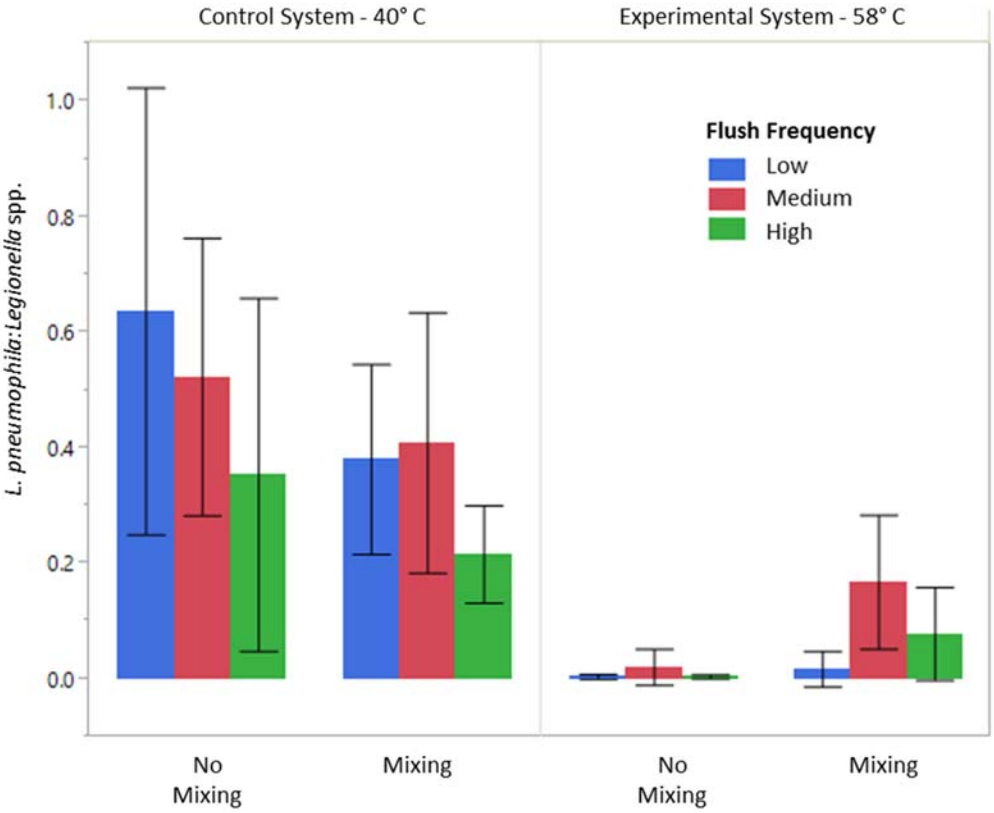

Figure S5. Relationship between the L. pneumophila to Legionella spp. ratio by system, convective mixing gradient, and use frequency.

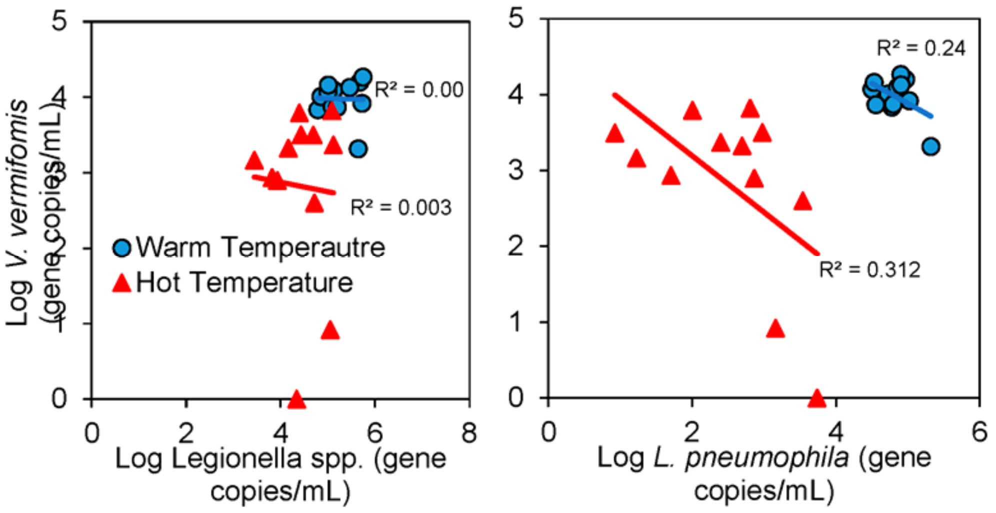

Figure S6. Correlation of *V. vermiformis* with (**A**) *Legionella* spp. and (**B**) *L. pneumophila* in distal tap bulk water.
